# Supplementary material for: The effect of magnetic field on chiral transmission in p-n-p graphene junctions
Source: Sci Rep. 2015 Dec 18;5:18458. doi: 10.1038/srep18458 (PMC4683455; doi:10.1038/srep18458)
Supplement: Supplementary Information [file srep18458-s1.pdf]

The effect of magnetic field on chiral transmission in p-n-p graphene junctions

Yuan Li[1],* Qi Wan[1], Yingzi Peng[1], Guanqing Wang[1],
Zhenghong Qian[2], Guanghui Zhou[3], and Mansoor B. A. Jalil[4†]

[1]*Department of Physics, Hangzhou Dianzi University, Hangzhou, Zhejiang 310018, China*

[2] *Center for Integrated Spintronic Devices (CISD), Hangzhou Dianzi University, Hangzhou, Zhejiang 310018, China*

[3]*Department of Physics and Key Laboratory for Low-Dimensional Quantum Structures and Manipulation (Ministry of Education), Hunan Normal University, Changsha 410081, China and*

[4]*Computational Nanoelectronics and Nano-device Laboratory, Electrical and Computer Engineering Department, National University of Singapore, 4 Engineering Drive 3, Singapore 117576, Singapore*

Abstract

We investigate Klein tunneling in graphene heterojunctions under the influence of a perpendicular magnetic field via the non-equilibrium Green's function method. We find that the angular dependence of electron transmission is deflected sideways, resulting in the suppression of normally incident electrons and overall decrease in conductance. The off-normal symmetry axis of the transmission profile was analytically derived. Overall tunneling conductance decreases to almost zero regardless of the potential barrier height V_0 when the magnetic field (B-field) exceeds a critical value, thus achieving effective confinement of Dirac fermions. The critical field occurs when the width of the magnetic field region matches the diameter of the cyclotron orbit. The potential barrier also induces distinct Fabry-Pérot fringe patterns, with a "constriction region" of low transmission when V_0 is close to the Fermi energy. Application of B-field deflects the Fabry-Pérot interference pattern to an off-normal angle. Thus, the conductance of the graphene heterojunctions can be sharply modulated by adjusting the B-field strength and the potential barrier height relative to the Fermi energy.

PACS numbers: 73.23.Ad, 73.40.Lq, 73.43.Qt, 81.05.Uw

*Electronic address: liyuan@hdu.edu.cn

†Electronic address: elembaj@nus.edu.sg

Introduction

Graphene consists of a one-atom-thick carbon honeycomb lattice and has attracted considerable interest in both experimental and theoretical studies [1, 2]. In graphene systems, the charge carriers possess a gapless linear dispersion in the low-energy approximation, which results in the particles behaving as massless Dirac fermions. Due to the chiral nature of the charge carriers, electrons injected at normal direction can transmit perfectly across an arbitrarily high and wide barriers, referred to as Klein tunneling [3, 4]. However, the peculiar Klein tunneling has become a double-edged sword. On the one hand, the perfect transmission suppresses the backscattering of charge carriers and protects the high mobility of the graphene system. On the other hand, Klein tunneling makes the confinement of the massless Dirac fermions difficult, especially the electrostatic confinement [5–7].

In the context of graphene device application, the requirement of effective confinement of the Dirac fermions, has triggered active research effort. Existing literatures have reported the possibility of confinement of Dirac fermions in quantum systems formed by electrostatic potentials, such as quantum dot islands [8–12], electron cloaks [13] or by gate geometry alone [14, 15]. Owing to the different chirality of the charge carriers, it is possible to utilize an electrical method to modulate the transport properties of Dirac fermions in few-layer graphene systems, for example twisted graphene bilayer [16], trilayer graphene [17–19]. For the case of monolayer graphene, it has been reported that inhomogeneous magnetic fields may be utilized to confine massless Dirac fermions [20], which motivates subsequent works based on wave vector filtering [21–23]. Based on the confining characteristic of the magnetic field, different magnetic configurations and magnetic graphene superlattices are proposed to control the transport property of the charge carriers in graphene systems [24–29]. In particular, the combined effect of applied magnetic field and electrostatic potential induces novel effects, such as the collapse of Landau levels [30, 31], Fabry-Pérot interference [32–34] and other interesting transport phenomena [35–40], upon transmission of Dirac fermions through the graphene junctions. **However, thus far, most research work had adopted the low-energy Dirac Hamiltonian to study the effect of the magnetic field on the graphene transport which neglects the contribution of higher-order terms in the energy dispersion, and is limited to simple configurations of the graphene junctions.** In addition, the effect of the magnetic field (B-field)on the Fabry-Pérot interference

pattern and hence, on the total conductance deserves further study.

Motivated by the above issues, in this paper, we consider a gated graphene junction influenced by external magnetic fields B_1 and B_2, and electrostatic potential V_0, as shown schematically in Fig. 1. We adopt the tight-binding model to analyze the transport property, which does not assume the low-energy Dirac approximation. Based on the nonequilibrium Green's function approach, we calculate the transmission and the total conductance under the influence of an applied magnetic field. We found that the angular dependence of transmission is deflected sideways by the magnetic B-field, resulting in the decrease of the total conductance. Correspondingly, the axis of symmetry of the transmission is shifted to an off-normal orientation, which can be analytically determined based on the B-field strength. The transmission is suppressed close to zero when the magnetic field increases beyond a critical field. Thus, one can effectively confine the Dirac fermions in the graphene device by either increasing the B-field or the width of the low magnetic-field region to accommodate a cyclotron orbit. The magnetic field induces also deflects the distinct Fabry-Pérot fringe patterns induced by the potential barrier, and can significantly decrease the electron transmission within a certain "constriction"' region.

The rest of this paper is organized as follows. **In the section of methodology, we present the tight-binding model and the transport formalism of the nonequilibrium Green's function method. In the section of results and discussion, we discuss the angle-dependent transmission and the total conductance under the influence of the magnetic field. Finally in the section of conclusions, we give a summary of our conclusions.**

Methodology

In this section, we consider the effect of the external magnetic field on the Klein tunneling of bulk graphene heterojunctions, as shown in Fig. 1. The scattering region includes red and blue regions, which are acted upon by two different magnetic fields, B_1 and B_2, respectively. We adopt the π-orbital tight-binding model to investigate the Klein tunneling under perpendicular magnetic fields. The real space π-orbital tight-binding Hamiltonian is

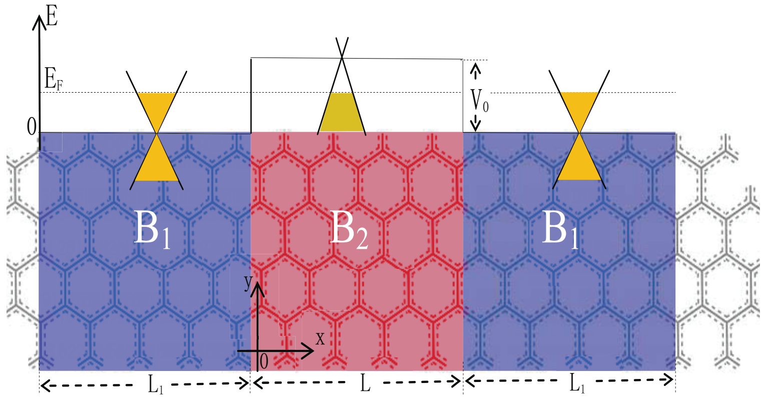

FIG. 1: Schematic of the gated graphene heterojunction structure modulated by external magnetic fields.

written as

$$H = \sum_i V_i c_i^\dagger c_i - t \sum_{\langle i,j \rangle} c_i^\dagger c_j, \tag{1}$$

where t is the hopping energy between two neighboring atoms i and j, $c_i^\dagger(c_i)$ refers to the creation (annihilation) operator, $V_i = V_0\Theta(x)\Theta(L-x)$ is the on-site potential at site i.

The orbital contribution of the external magnetic field perpendicular to the graphene plane is introduced through the Peierls substitution of hopping coefficients [41],

$$t \to t\ \exp(\frac{-ie}{\hbar}\int_i^j d\mathbf{l}\cdot\mathbf{A}). \tag{2}$$

In our tight-binding method we adopt the Bloch theorem along the transverse direction with periodicity l_y [42]. For the bulk graphene oriented with zigzag carbon chains along the x direction, we can choose the transverse periodicity $l_y = 3a_0$ to effectively describe the bulk graphene system, where $a_0 \approx 1.42$ Å is the bonding length. We adopt the Landau gauge $\mathbf{A} = (0, -xB_z, 0)$ in the scattering region to maintain the transverse translation invariance throughout the whole system. Thus the wave vector k_y is still a good quantum number, so that the Bloch theorem can still be used to model the transport induced by the magnetic field. The Bloch treatment simplifies the quantum transport model of the large-area graphene system to be studied because one just needs to analyze a graphene nanoribbon with zigzag chain number of $N_y = 2$, thus significantly reducing the matrix size involved in the numerical computation. **The sites at the transverse boundaries are**

wrapped around and so as to overlap with each other, thus inducing an extra hopping term for these two sites. Applying the periodic boundary conditions, we introduced a Bloch phase factor $e^{ik_y l_y}$ to modify these hopping terms with k_y being the Bloch wave vector [43]. The Bloch momentum k_y is defined as $k_y = k_F \sin\theta$, where $k_F = 2E_F/(3a_0 t)$ denotes the Fermi wave vector [2] and θ is the incident angle of the electrons.

We calculate the transmission probability for an electron injected with Fermi energy E_F and wave vector k_y across the graphene heterojunction in terms of the Fisher-Lee relation [43, 44]

$$T(k_y, E_F) = \mathrm{Tr}(\Gamma_L G^r \Gamma_R G^a), \tag{3}$$

where $\Gamma_\alpha = i[\Sigma_\alpha^r - \Sigma_\alpha^a]$ is the line-width function, and Σ_α^r is the retarded self-energy function coupling to the leads $\alpha = L,\ R$, which can be calculated numerically by solving the surface Green's function of the leads [45]. **If the line-width function Γ_α is obtained, we can express the retarded and advanced Green's functions according to their respective definitions**

$$G^r(E) = [G^a(E)]^\dagger = [E - H_c - \Sigma_L^r - \Sigma_R^r]^{-1}, \tag{4}$$

with H_c being the Hamiltonian of the central scattering region. **Note that the length of the scattering region is $L + 2L_1$, where the value of L_1 is taken to be zero in the absence of the magnetic field B_1. In our calculations, we consider a graphene nanoribbon with zigzag chain number of $N_y = 2$ (i.e., of transverse width $3a_0$) but with a periodic boundary condition to mimic a graphene sheet of infinite transverse width.**

The electron is injected into the central region at various incidence angle θ, associated with different values of k_y. We calculate the transmission probability for a fixed k_y or θ, i.e., $T(\theta, E_F)$. Then the conductance of the graphene heterojunction is obtained by integration of Eq. (3) over the transverse Bloch momentum [35],

$$G = \frac{2e^2}{h}\frac{W}{2\pi}\int dk_y T(k_y, E_F) = G_0 \int_{-\pi/2}^{\pi/2} d\theta \cos\theta T(\theta, E_F), \tag{5}$$

where the factor of 2 accounts for the two-fold spin degeneracy, W is the transverse dimension

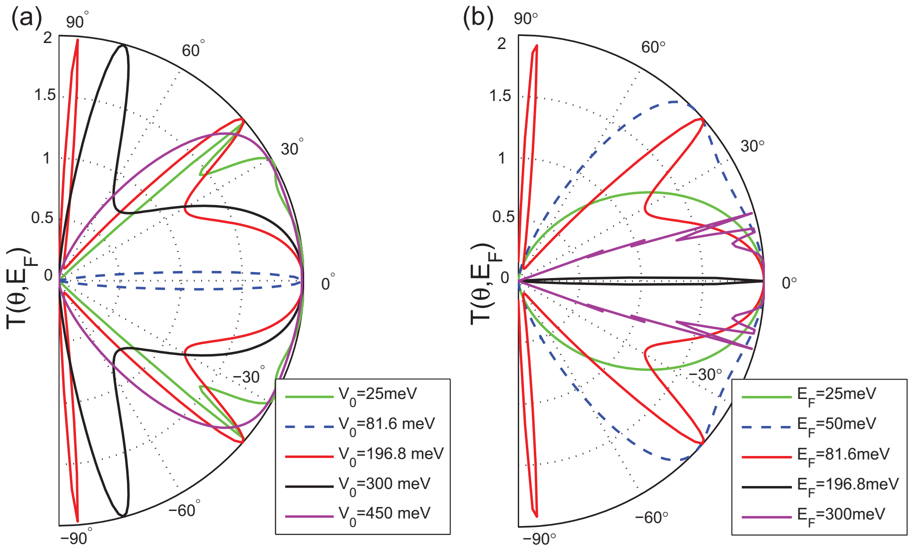

FIG. 2: The total transmission $T(\theta, E_F)$ plotted as a function of the electron injection-angle θ for (a) $E_F = 81.6$ meV, (b) $V_0 = 196.8$ meV. In both cases the barrier width is $L = 100$ nm, the hopping energy is $t = 3$ eV and $B_1 = B_2 = 0$.

of the interface and $G_0 = e^2 k_F W/(\pi h)$ is the unit of conductance. Note that the valley degeneracy is inherently incorporated in the Hamiltonian H.

Results and Discussion

In this section, we first analyze the energy-dependent behavior of the transmission probability $T(\theta, E_F)$ in the absence of the magnetic field, i.e., $B_1 = B_2 = 0$. We plot $T(\theta, E_F)$ as a function of the incident angle θ for different Fermi energies and potential barriers V_0, as shown in Fig. 2. Fig. 2(a) shows similar angular dependence as that of Fig. 2 in Ref. [3] when $V_0 = 196.8,\ 300$ meV, obtained via the low-energy Dirac equation. It can be seen that the transmission can reach the maximum value of 2 even when the potential barrier is much higher than the Fermi energy (e.g., for $V_0 = 450$ meV), which is a typical signature of Klein tunneling. However, there is significant a suppression of electron transmission for the special case of $V_0 = E_F = 81.6$ meV, which we name as the equal-barrier point. For this case, although the transmission of normally incident electrons still remains at the ideal value of 2, there is a sharp decrease in T to almost zero when the angle of incidence deviates slightly from the normal. This phenomenon may be explained by considering the effective

theory of the low-energy approximation [2]. **Note that all the results presented in the figures in this paper are calculated numerically by means of the tight-binding non-equilibrium Greens function approach. The low-energy Dirac Hamiltonian is only used to obtain analytical results in order to elucidate the numerical results.** Accordingly, the Dirac equation of the graphene heterojunction can be written as

$$-iv_F\vec{\sigma}\cdot\nabla\psi(\mathbf{r})+V_0\psi(\mathbf{r})=E\psi(\mathbf{r}), \tag{6}$$

where v_F is the Fermi velocity, the operator $\nabla=(\partial_x,\partial_y)$ denotes the differential operator in the x-y plane, and $\psi(\mathbf{r})=[\varphi_A(x),\varphi_B(x)]e^{ik_yy}$ refers to the two-component wave function. Bearing in mind the potential profile of our tight-binding model, we can obtain the explicit forms of the wave functions for three regions at $E=V_0$ as follows,

$$\psi_I(\mathbf{r})=\frac{e^{ik_yy}}{\sqrt{2}}\left\{\begin{pmatrix}1\\ e^{i\theta}\end{pmatrix}e^{ik_xx}+r\begin{pmatrix}1\\ -e^{-i\theta}\end{pmatrix}e^{-ik_xx}\right\}, \tag{7}$$

$$\psi_{II}(\mathbf{r})=\frac{e^{ik_yy}}{\sqrt{2}}\begin{pmatrix}ae^{k_yx}\\ be^{-k_yx}\end{pmatrix}, \tag{8}$$

$$\psi_{III}(\mathbf{r})=\frac{t_0e^{ik_yy}}{\sqrt{2}}\begin{pmatrix}1\\ e^{i\theta}\end{pmatrix}e^{ik_xx}, \tag{9}$$

where r and t_0 refer to the reflection and transmission amplitudes, respectively. By imposing the matching conditions at the two interfaces, we obtain the transmission probability as

$$T=|t_0|^2=\frac{4\cos^2\theta}{[e^{2k_yL}+e^{-2k_yL}+2\cos(2\theta)]}. \tag{10}$$

Obviously, $T=1$ at $\theta=0$ ($k_y=0$), where the valley degeneracy has been neglected. However, the transmission rapidly decreases with increasing k_y or θ, especially for thick barrier region (i.e., large L), due to the exponential factor e^{2k_yL} in the denominator. Thus, one can deduce that for the special equal-barrier case, the transmission T is nonzero (and equals to 1) only at normal incidence, i.e. $\theta=0$, if the barrier region is sufficiently thick. This phenomena is similar to that of the two-dimensional dice lattice [46]. The same behavior is illustrated in Fig. 2(b), where the potential barrier is fixed at $V_0=196.8$ meV, while E_F is varied. Strong suppression of the transmission T again occurs at the equal-barrier point,

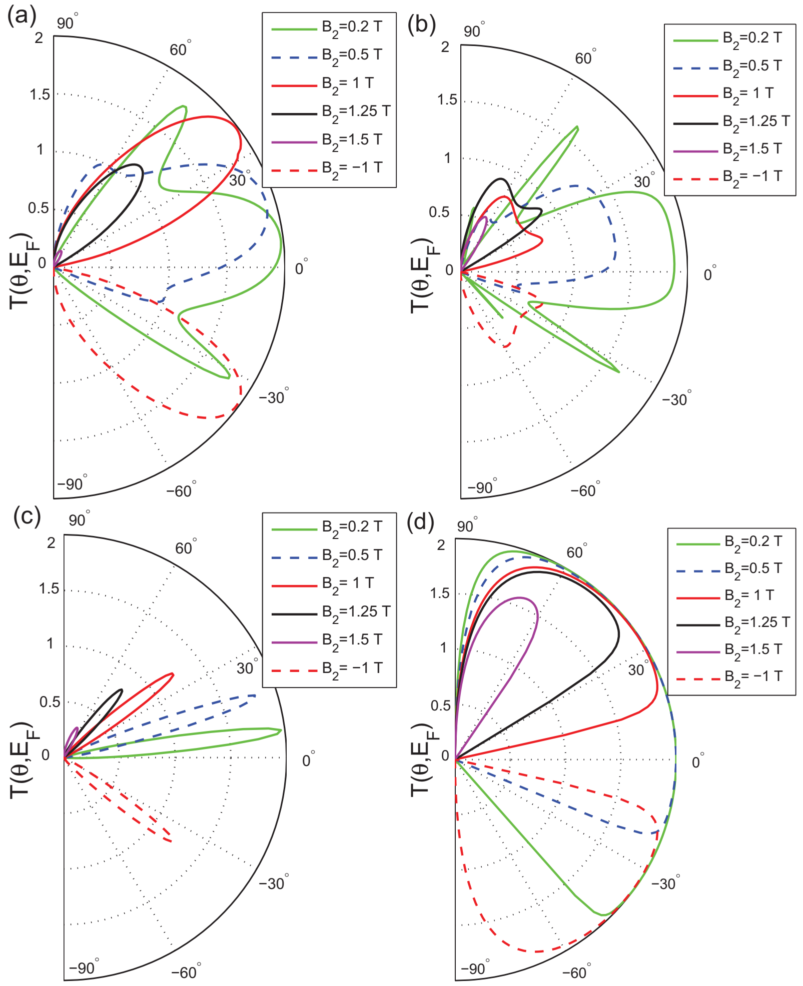

FIG. 3: The total transmission $T(\theta, E_F)$ plotted as a function of the electron injection-angle θ for different magnetic fields and potential barriers (a) $V_0 = 196.8$ meV, (b) $V_0 = 163.2$ meV, (c) $V_0 = 81.6$ meV, and (d) $V_0 = 0$. We choose $B_2 = -1$ T as an example of the B-field effect on the transmission applied with an opposite direction. The Fermi energy is fixed at $E_F = 81.6$ meV, $B_1 = 0$, and the other parameters are the same as those in Fig. 2.

namely $E_F = 196.8$ meV (black curve). It is interesting to note that the characteristic of Klein tunneling (i.e., perfect transmission for normally incident electrons) always holds regardless of the Fermi energy or the potential barrier height.

Next we analyze the effect of the magnetic field on the transmission. The transmission T is plotted in Fig. 3 versus the incident angle θ for different strengths of the magnetic field B_2 in the central region and potential barrier heights V_0, while B_1 is set to zero. Fig. 3 shows that the transmission profiles are deflected to one side. **Accordingly, the axis of**

symmetry is shifted from normal incidence ($\theta = 0$) to some off-normal angle θ_s, which satisfies the relation $\sin\theta_s = eB_2L/(2\hbar k_F)$ derived from the symmetry center of the Fabry-Prot pattern, as shown in our subsequent discussion on the Fabry-Pérot interference. With increasing strength of the magnetic field, e.g., for $B_2 = 1$ T [red curve in Fig. 3(a)], the transmission profile is confined to a single lobe of limited angular range of $25° < \theta < 65°$. In addition, normally incident electrons are completely reflected, resulting in the suppression of the Klein tunneling. When the B-field strength is increased further to $B_2 = 1.5$ T, the transmission lobe dramatically shrinks in size. Thus, transmission is suppressed for all angles of incidence resulting in much reduced overall conductance. It is thus natural to obtain a critical B-field beyond which the overall transmission is suppressed to zero. The critical field can be understood as the competition between the Lorentz force and the repulsive potential barrier [30]. **The shrinking of the transmission lobe can be understood from the semiclassical picture of the Lorentz force. In the presence of a perpendicular magnetic field, the Dirac fermions move along a circular cyclotron orbit with the cyclotron radius being $r_c = |E_F - V_0|/(ev_FB)$ [47]. With increasing magnetic field strength, the cyclotron radius becomes smaller, and it is thus easier for the Dirac fermions to complete a cyclotron orbit motion, resulting in a decrease of the transmission. From this point of view, only the electrons with some specific incident angles can transmit through the scattering region, and this is reflected by a smaller and narrower (in terms of angular spread) transmission lobe.** The existence of the critical field provides an avenue to confine the massless Dirac fermions since transmission is almost completely suppressed beyond the critical field.

Note that the critical field also distinguishes the quantum Hall regime and the low-field regime. In the former, application of strong magnetic fields leads to the formation of discrete Landau levels. However, when the magnetic field strength is decreased below the critical field, the Landau levels undergo a collapse transition [30, 31]. At field strength below this transition, the classical Lorentz force behavior is observed where the transmission profile is pushed sideways as described above. This means the suppression of transport, as can be seen by calculating the conductance with Eq. (5). In particular, the transmission approaches zero when the magnetic field is close to the critical field, a feature which can be utilized to confine the Dirac fermions in a graphene system via the suppression of Klein tunneling. However, in

the quantum Hall regime, such confinement is not possible. This is because the Landau level states induce edge transport [48, 49], thus contributing to a non-zero conductance. Thus, we limit our investigation of the magnetic field-induced transport to the low-field regime, to avoid the contribution of the edge states and achieve an effective confinement of Dirac fermions in graphene.

Next, we analyze the transport behavior induced by the magnetic field for the special equal-barrier case, i.e., $E_F = V_0 = 81.6$ meV [Fig. 3(c)]. The curves of the transmission are also deflected to one side, however, the transmission lobes are much sharper compared to other potential barrier heights. This shows that the strong suppression of transmission in the equal-barrier case, as shown in Fig. 2 (in the absence of magnetic field), still holds under application of B-field. The sharpness of the transmission lobes and the ability to deflect them via a B-field indicates the possibility of strong transport modulation by varying the magnetic field strength. We also consider the transport behavior when the magnetic field direction is reversed to the $+\hat{z}$ direction. As shown, e.g., by the transmission curve corresponding to $B = -1$ T in Fig. 3(c), only electrons with negative incident angle can transmit through the barrier. In other words, the transmission lobe is deflected by the same angle as the case of $B = 1$ T, but in the opposite direction.

For comparison with the results of a gated graphene junction (i.e., with a finite potential V_0), we calculate the angle-dependent transmission in the absence of the potential barrier, i.e., $V_0 = 0$, as illustrated in Fig. 3(d). The transmission lobes span over a wider angular range. The transmission profiles also lack the subsidiary lobes that are generally observed in the case of finite V_0 [see Figs. 3(a) and (b)], which are associated with the Fabry-Pérot interference. It is also observed that the decreasing trend of the transmission with increasing magnetic field is less pronounced, compared with the finite V_0 case. Thus, for the effective confinement of the Dirac electrons, it is advantageous to have a gated or n-p-n graphene junction to induce an electrostatic barrier, rather than a graphene sheet with only a magnetic barrier. Regardless of the barrier height, the transmission approaches zero when B_2 exceeds 1.5 T for all incident angles [see Figs. 3(a) to (d)]. According to the low-energy theory [20], there is zero transmission beyond a critical B-field such that the condition $L_c \leq L$, where L is the width of the B-field region and L_c is the diameter of the cyclotron orbit in monolayer graphene, which is given by $L_c = 2E_F l_B^2/\hbar v_F$, with $l_B = \sqrt{\hbar/eB_2} \approx 21$ nm, being the magnetic length. For the width L=100 nm and $E_F = 81.6$ meV considered in our

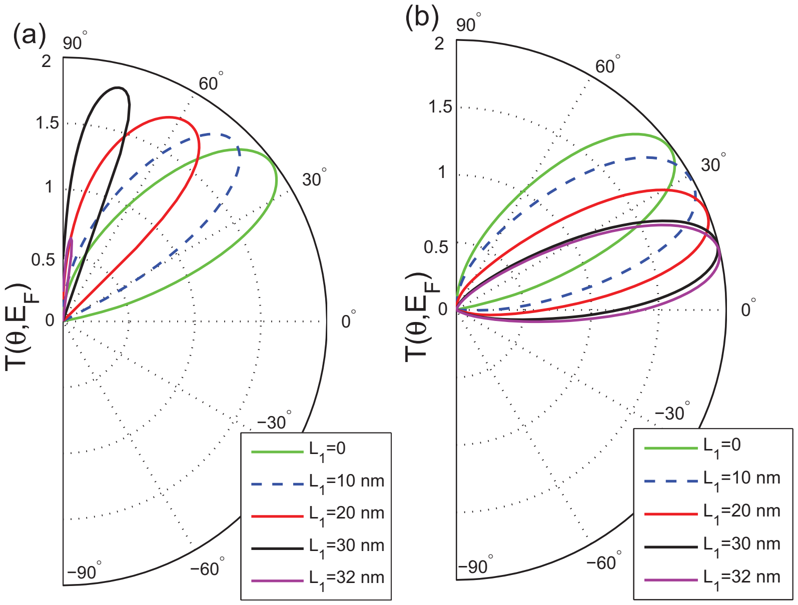

FIG. 4: The total transmission $T(\theta, E_F)$ plotted as a function of the electron injection-angle θ for different lengths L_1 for (a) $B_2 = B_1 = 1$ T and (b) $B_2 = -B_1 = 1$ T. The Fermi energy is $E_F = 81.6$ meV, $V_0 = 196.8$ meV, $L = 100$ nm, and other parameters are the same as those in Fig. 2.

calculations, the critical field turns out to be $B_2 \approx 1.62$ T, which agrees with our calculated results.

We now consider the situation where the magnetic field is set at a sub-critical value to avoid the existence of the edge states, while the length of the magnetic field region is increased to effectively confine the Dirac electrons. As shown in Fig. 1, we included two additional regions of length L_1, on either side of the central region, where a magnetic field B_1 is applied. Fig. 4(a) shows the angle-dependent transmission for different lengths L_1 when $B_2 = B_1 = 1$ T. With the length of L_1 increasing from 0 to 32 nm, the transmission lobe gets pushed gradually towards the transverse y direction, and overall transmission decreases to zero. The critical length where T goes to zero is at about $L_1 = 32$ nm, in agreement with the cyclotron diameter at $B = 1$ T. If the direction of B_1 is reversed to the $+\hat{z}$ direction, we find that the transmission lobes are gradually pushed back towards the direction $\theta = 0$ [see Fig. 4(b)]. This means that a negative magnetic field B_1 will offset the effect of B_2 and recover the Klein tunneling characteristic, resulting in the increase of the conductance.

Fig. 5 shows the contour plots of the transmission as a function of the potential barrier

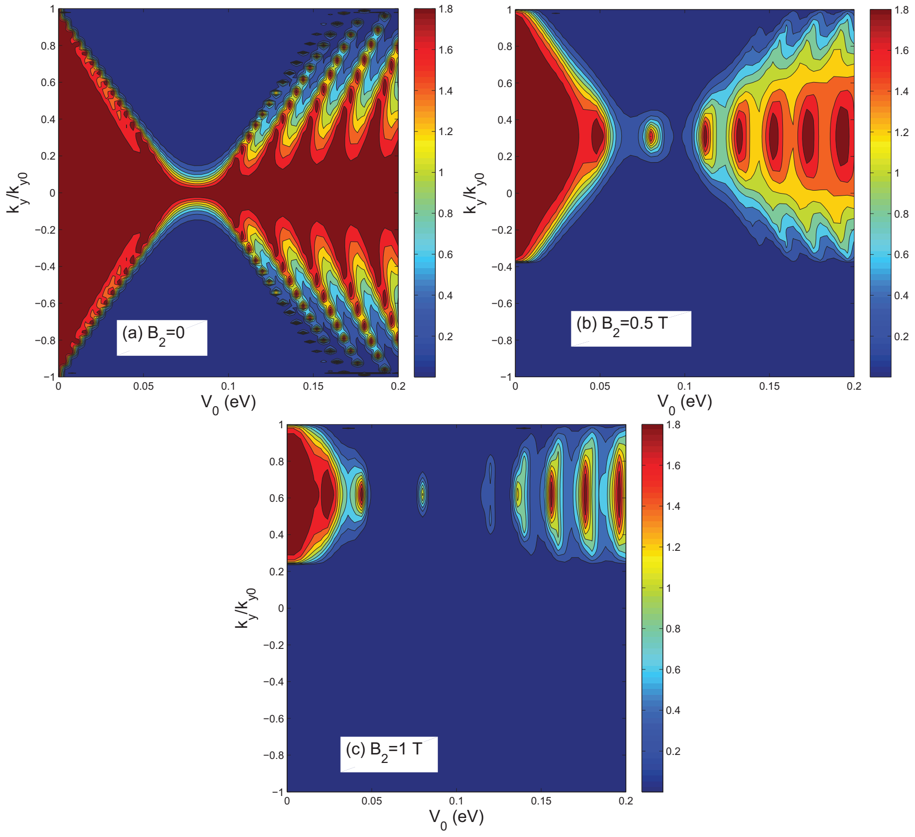

FIG. 5: Contour plot of the transmission of the *n*-*p*-*n* graphene heterojunction as a function of the potential barrier V_0 and the wave vector k_y for different magnetic field stregths: (a) $B_2 = 0$, (b) $B_2 = 0.5$ T, and (c) $B_2 = 1$ T. The parameter $k_{y0} = k_F$, while $E_F = 80$ meV, $B_1 = 0$, and other parameters are the same as those in Fig. 2.

V_0 and the transverse wave vector k_y for different magnetic fields B_2. In this numerical calculation, B_1 is set to zero and $E_F = 80$ meV. When $B_2 = 0$, electrons at normal incidence undergo perfect transmission through the graphene junction irrespective of the value of V_0, which reflects the characteristic of Klein tunneling. It is obvious that the transmission contour plot is symmetrical about $k_y = 0$. Interestingly, there exists a constriction centered at $V_0 = E_F = 80$ meV, the equal-barrier point. This constriction feature translates to the sharp transmission lobe observed in the special equal-barrier case, as shown in Fig. 2. At finite magnetic field, e.g., $B_2 = 0.5$ T, the axis of symmetry of the transmission plot is shifted upwards, and coincides with the value $k_y/k_{y0} = eB_2L/(2\hbar k_F) \approx 0.31$. This translates to the deflection of the transmission lobe to the angle corresponding to $\sin\theta_s = eB_2L/(2\hbar k_F)$ in

the presence of the B-field. To derive the relation governing the symmetry axis, we consider the Fabry-Pérot fringe pattern, which arises from the interference of the scattering on the two n-p interfaces.

According to the low-energy theory, the Fabry-Pérot interference can be attributed to the Landau-Zener-like transition, by regarding the Dirac equation as a time-dependent Schrodinger equation of a two-level system, and treating the spatial coordinate x as time variable [32]. According to this analogy, a finite level splitting $-2i(k_y - eB_2x/\hbar)$ at the crossing points x =0, L can induce constructive or destructive interference of a superposition of states, resulting in the Fabry-Pérot pattern. Accordingly, the mid-point between the crossing points $x = L/2$, yields the symmetry center of the Fabry-Pérot pattern, i.e., $k_y = eB_2L/2\hbar$, which correspond to the above relation satisfied by θ_s. The location of the Fabry-Pérot fringes can be obtained by considering the special phase values of $\Delta\vartheta = -2k_F \int_0^L V_0 dx/E_F = 2n\pi$ ($n = 1, 2, ...$), which correspond to $V_{0n} = n\pi E_F/k_F L$, in agreement with the observed patterns in Fig. 5 (b)-(c). Finally, one can see that the transmission is especially suppressed with increasing B-field when V_0 is close to the constriction or equal-barrier region. This means that one can achieve a greater degree of B-field modulation of the conductance through the graphene heterojunction by adjusting the potential barrier height or the Fermi energy, so as to coincide with the constriction region.

Our tight-binding model with a sharp potential barrier yields similar transport behaviors with that obtained via the low-energy theory with a smooth potential, which is studied in Ref. [32]. However, surprisingly, the contour plot of the transmission in Ref. [32] remains symmetrical about $p_y = 0$, and not deflected to one side by the magnetic field. In order to understand why this difference arises, we calculate the transmission of the graphene heterojunction with a slightly different magnetic field profile: $B_2(x) = B_2\Theta(L/2 - |x|)$. In other words, the origin of the coordinate x is shifted to the center of the potential barrier. For this symmetrical magnetic configuration, the corresponding vector potential profile is given by

$$A(x) = -B_2 \times \begin{cases} -L/2, & x < -L/2 \\ x, & |x| \leq L/2 \\ L/2, & x > L/2 \end{cases} \tag{11}$$

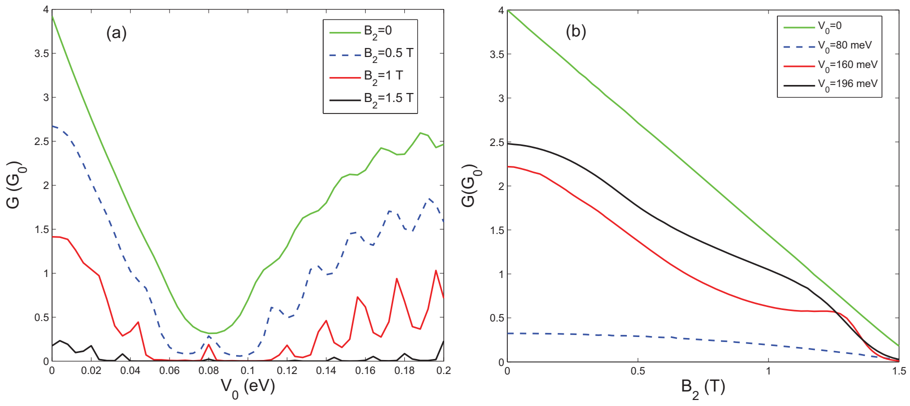

FIG. 6: The conductance G plotted as a function of (a) the potential barrier V_0 and (b) the strength of the magnetic field. The Fermi energy is fixed at $E_F = 80$ meV, $B_1 = 0$, and the other parameters are the same as those in Fig. 2.

This gauge choice requires the wave vector to be modified as

$$k_x = k_F \cos\theta, \qquad k_y = k_F \sin\theta - eB_2L/2\hbar, \tag{12}$$

in order to preserve the gauge invariance of the transmission [20] and to ensure continuity of the magnetic vector potential across the two junctions. Therefore, with the modified wave vector, we obtain the similar results with those shown in Fig. 5 for the symmetrical magnetic configuration. In Ref. [32], there is no sideway deflection in the transmission profile because they are plotting the canonical momentum, while we focus on the kinematic momentum which would reflect the trajectory of the Dirac fermions within the magnetic region.

Finally, we analyze the effect of the field on the conductance G. Fig. 6(a) shows the conductance as a function of the potential barrier V_0 for different magnetic fields. When $B_2 = 0$, the conductance decreases as V_0 is increased, from a value of 4 in reduced units at $V_0 = 0$ to a minimum value of $G \approx 0.4$ at the equal-barrier point where $V_0 = E_F = 80$ meV [corresponding to the center of the constriction region shown in Fig. 5(a)], and then gradually increases to about 2.5. In general, G can be significantly decreased by increasing the magnetic field B_2. For example $B_2 = 1$ T (red curve), the conductance is suppressed to virtually zero within the regime $55 < V_0 < 110$ meV, except for a cusp around the equal-barrier point. The small cusp can be attributed to the contribution of the Fabry-Pérot fringe shown in Fig. 5(c). For the case of a n-p-n graphene junction, i.e., $V_0 > E_F$, the conductance exhibits distinct peaks associated with the Fabry-Pérot oscillations. Fig. 6(b)

shows the variation of the conductance as a function of the magnetic field for different potential barrier heights. We can see that the conductance decreases almost monotonically to close to zero as B_2 is increased. For all magnetic field strengths, the conductance is smallest at the equal-barrier point, which again demonstrates that the best confinement of Dirac fermions in the graphene junction occurs in the constriction region. When $B_2 \approx 1.5$ T (close to the critical field value), all of the conductance curves approach zero, regardless of the potential barrier height. Thus, at this field value, the Dirac fermions are effectively confined by the formation of complete cyclotron orbit, as discussed previously.

Conclusions

In summary, we have investigated the transport property of a gated graphene sheet in the presence of magnetic fields using the non-equilibrium Green's function method. Our numerical calculations demonstrate the effect of the magnetic field on the angular dependence of the transmission and the total conductance. In the absence of the magnetic field, we find that the transmission is significantly suppressed when the potential barrier is equal to the Fermi energy. However, the characteristic of the Klein tunneling (perfect transmission for normally incident electrons) always holds regardless of the potential barrier height and the Fermi energy value. Upon application of an external perpendicular magnetic field on the graphene sheet, the angular-dependent transmission profile is deflected to one side, resulting in the decrease of the overall conductance. Accordingly, the orientation of the axis of symmetry of the transmission profile is shifted from the zero angle (normal incidence) to a certain off-normal angle θ_s, which satisfies a relation arising from the effect of the Fabry-Pérot interference. In the presence of a sufficiently strong B-field, normally incident electrons are almost completely reflected, resulting in the suppression of the Klein tunneling effect. When the B-field exceeds a certain critical value, the transmission is virtually zero regardless of the angle of incidence. This critical field value is associated with the formation of a complete cyclotron orbit, and marks the boundary between the quantum Hall regime and the low-field regime. Since we are interested in achieving effective confinement of Dirac fermions in graphene, we limit our analysis to the low-field regime, so as to avoid conductance contribution from the edge states in the quantum Hall regime. For a gated graphene sheet, one can effectively decrease the transmission through increasing the width of the magnetic-field

region. Interestingly, one can recover the characteristic of Klein tunneling **by applying** an additional magnetic field outside the central region, but in the opposite orientation. The contour plots of transmission as a function of k_y and V_0 show distinct Fabry-Pérot interference pattern whose fringe location agrees with analytical result. The transmission profile and the Fabry-Pérot pattern experience sideway deflection due to the effect of the magnetic field. The axis of symmetry upon deflection is the same as that obtained earlier from the angular-dependent transmission profile. We find that the transmission is reduced near the constriction (equal-barrier) region with increasing magnetic field. Thus, one can effectively control the conductance of the graphene heterojunction by adjusting the height of the potential barrier or the Fermi energy to coincide with the constriction region. Finally, the above transport characteristics are also reflected in the overall conductance, obtained by integrating the transmission over the incident angle.

---

[1] Beenakker, C. W. J. Colloquium: Andreev reflection and Klein tunneling in graphene. *Rev. Mod. Phys.* **80**, 1337-54 (2008);

[2] Castro Neto, A. H., Guinea, F., Peres, N. M. R., Novoselov, K. S. & Geim, A. K. The electronic properties of graphene. *Rev. Mod. Phys.* **81**, 109-162 (2009).

[3] Katsnelson, M. I., Novoselov, K. S. & Geim, A. K. Chiral tunnelling and the Klein paradox in graphene. *Nat. Phys.* **2**, 620-625 (2006).

[4] Cheianov, V. V., Faĺko, V. & Altshuler, B. L. The Focusing of Electron Flow and a Veselago Lens in Graphene p-n Junctions. *Science* **315**, 1252-55 (2007).

[5] Chen, H. Y., Apalkov, V. & Chakraborty, T. Fock-Darwin States of Dirac Electrons in Graphene-Based Artificial Atoms. *Phys. Rev. Lett.* **98**, 186803 (2007).

[6] Hewageegana, P. & Apalkov, V. Electron localization in graphene quantum dots. *Phys. Rev. B* **77**, 245426 (2008).

[7] Matulis, A. & Peeters F. M. Quasibound states of quantum dots in single and bilayer graphene. *Phys. Rev. B* **77**, 115423 (2008).

[8] Silvestrov, P. G. & Efetov, K. B. Quantum Dots in Graphene. *Phys. Rev. Lett.* **98**, 016802 (2007).

[9] Stampfer, C. *et al.* Tunable Coulomb blockade in nanostructured graphene. *Appl. Phys. Lett.*

92, 012102 (2008).

[10] Ponomarenko, L. A. *et al.* Chaotic dirac billiard in graphene quantum dots. *Science* **320**, 356-358 (2008).

[11] Güttinger, J. *et al.* Electron-Hole Crossover in Graphene Quantum Dots. *Phys. Rev. Lett.* **103**, 046810 (2009).

[12] Moriyama, S. *et al.* Coupled Quantum Dots in a Graphene-Based Two-Dimensional Semimetal. *Nano Lett.* **9**, 2891-2896 (2009).

[13] Liao, B., Zebarjadi, M., Esfarjani, K. & Chen, G. Isotropic and energy-selective electron cloaks on graphene. *Phys. Rev. B* **88**, 155432 (2013).

[14] Sajjad, R. N., Sutar, S., Lee, J. U. & Ghosh, A. W. Manifestation of chiral tunneling at a tilted graphene p-n junction. *Phys. Rev. B* 86, 155412 (2012).

[15] Sajjad, R. N. & Ghosh, A. W. Manipulating Chiral Transmission by Gate Geometry: Switching in Graphene with Transmission Gaps. *ACS Nano* **7**, 9808-9813 (2013).

[16] He, W. Y., Chu, Z. D. & He, L. Chiral Tunneling in a Twisted Graphene Bilayer. *Phys. Rev. Lett.* **111**, 066803 (2013).

[17] Lui, C. H., Li, Z. Q., Mak, K. F., Cappelluti, E. & Heinz, T. F. Observation of an electrically tunable band gap in trilayer graphene. *Nat. Phys.* **7** 944-947 (2011).

[18] Kumar, S. B. & Guo, J. Chiral tunneling in trilayer graphene. *Appl. Phys. Lett.* **100**, 163102 (2012).

[19] Duppen, B. V., Sena, S. H. R. & Peeters, F. M. Multiband tunneling in trilayer graphene. *Phys. Rev. B* **87**, 195439 (2013).

[20] Martino, A. D., Dell'Anna, L. & Egger, R. Magnetic Confinement of Massless Dirac Fermions in Graphene. *Phys. Rev. Lett.* **98**, 066802 (2007).

[21] Masir, M. R., Vasilopoulos, P. & Peeters, F. M. Wavevector filtering through single-layer and bilayer graphene with magnetic barrier structures. *Appl. Phys. Lett.* **93**, 242103 (2008).

[22] Masir, M. R., Vasilopoulos, P., Matulis, A. & Peeters, F. M. Direction-dependent tunneling through nanostructured magnetic barriers in graphene. *Phys. Rev. B* **77**, 235443 (2008).

[23] DellAnna, L. & Martino, A. D. Wave-vector-dependent spin filtering and spin transport through magnetic barriers in graphene. *Phys. Rev. B* **80**, 155416 (2009).

[24] DellAnna, L. & Martino, A. D. Multiple magnetic barriers in graphene. *Phys. Rev. B* **79**, 045420 (2009).

[25] Wu, Q. S., Zhang, S. N. & Yang, S. J. Transport of the graphene electrons through a magnetic superlattice. *J. Phys.: Cond. Matt.* **20**, 485210 (2008).

[26] Low, T. & Appenzeller, J. Electronic transport properties of a tilted graphene p-n junction. *Phys. Rev. B* 80, 155406 (2009).

[27] Yan, H. *et al.* Superlattice Dirac points and space-dependent Fermi velocity in a corrugated graphene monolayer. *Phys. Rev. B* **87**, 075405 (2013).

[28] Sattari, F. & Faizabadi, E. Spin transport and wavevector-dependent spin filtering through magnetic graphene superlattice. *Solid State Comm.* **179**, 48-53 (2014).

[29] Zhai, F. & Yang, L. Strain-tunable spin transport in ferromagnetic graphene junctions. *Appl. Phys. Lett.* **98**, 062101 (2011).

[30] Gu, N., Rudner, M., Young, A., Kim, P. & Levitov, L. Collapse of Landau Levels in Gated Graphene Structures. *Phys. Rev. Lett.* **106**, 066601 2011.

[31] Lukose, V., Shankar, R. & Baskaran, G. Novel Electric Field Effects on Landau Levels in Graphene. *Phys. Rev. Lett.* **98**, 116802 (2007).

[32] Shytov, A. V., Rudner, M. S. & Levitov, L. S. Klein Backscattering and Fabry-Pérot Interference in Graphene Heterojunctions. *Phys. Rev. Lett.* **101**, 156804 (2008).

[33] Shytov, A., Rudner, M., Gu, N., Katsnelson, M. & Levitov, L. Atomic collapse, Lorentz boosts, Klein scattering, and other quantum-relativistic phenomena in graphene. *Solid State Comm.* 149, 1087-1093 (2009).

[34] Masir, M. R., Vasilopoulos, P. & Peeters, F. M. Fabry-Pérot resonances in graphene microstructures: Influence of a magnetic field. *Phys. Rev. B* **82**, 115417 (2010).

[35] Cheianov, V. V. & Fal'ko, V. I. Selective transmission of Dirac electrons and ballistic magnetoresistance of n-p junctions in graphene. *Phys. Rev. B* **74**, 041403(R) (2006).

[36] Huard, B. *et al.* Transport Measurements Across a Tunable Potential Barrier in Graphene. *Phys. Rev. Lett.* **98**, 236803 (2007).

[37] Song, Y. & Guo, Y. Electrically induced bound state switches and near-linearly tunable optical transitions in graphene under a magnetic field. *J. Appl. Phys.* **109**, 104306 (2011).

[38] Moriyama, S., Morita, Y., Watanabe, E. & Tsuya, D. Field-induced confined states in graphene. *Appl. Phys. Lett.* **104**, 053108 (2014).

[39] Kumar, S. B., Jalil, M. B. A. & Tan, S. G. Klein tunneling in graphene systems under the influence of magnetic field. *J. Appl. Phys.* **114**, 084314 (2013).

[40] **Li, Y., Jalil, M. B. A. & Zhou, G. H. Giant magnetoresistance modulated by magnetic field in graphene p-n junction. *Appl. Phys. Lett.* 105, 193108 (2014).**

[41] Peierls, R. On the theory of diamagnetism of conduction electrons. *Z. Phys. A* **80**, 763-791 (1933).

[42] Liu, M. H., Bundesmann, J. & Richter, K. Spin-dependent Klein tunneling in graphene: Role of Rashba spin-orbit coupling. *Phys. Rev. B* **85**, 085406 (2012).

[43] Datta, S. Quantum transport: atom to transistor (Cambridge: Cambridge University Press). *p. 109* (2005).

[44] Fisher, D. S. & Lee, P. A. Relation between conductivity and transmission matrix. *Phys. Rev. B* **23**,6851 (1981).

[45] Sancho, M. P. L., Sancho, J. M. L. & Rubio, J. Highly convergent schemes for the calculation of bulk and surface Green functions. *J. Phys. F: Met. Phys.* **15**, 851-858 (1985).

[46] Urban, D. F., Bercioux, D., Wimmer, M. & Häusler, W. Barrier transmission of Dirac-like pseudospin-one particles. *Phys. Rev. B* **84**, 115136 (2011).

[47] Rakhimov, K. Y., Chaves, A., Farias, G. A. & Peeters, F. M. Wavepacket scattering of Dirac and Schrödinger particles on potential and magnetic barriers. *J. Phys.: Condens. Matter* **23**, 275801 (2011).

[48] Abanin, D. A. & Levitov, L. S. Quantized Transport in Graphene p-n Junctions in a Magnetic Field. *Science* **317**, 641-643 (2007).

[49] Williams, J. R., DiCarlo, L. & Marcus, C. M. Quantum Hall Effect in a Gate-Controlled p-n Junction of Graphene. *Science* **317**, 638-641 (2007).

Acknowledgments

This work was supported by the National Natural Science Foundation of China (Grants No. 11204058 and 11574067), Natural Science Foundation of Zhejiang Province (Grant No. LY16A040007), and the National Research Foundation of Singapore under the Competitive Research Program "Non-Volatile Magnetic Logic And Memory Integrated Circuit Devices" NRF-CRP9-2011-01.

Author contributions

Y.L. conceived the idea and contributed to the theoretical analysis and interpretation of data, and wrote the manuscript. M.B.A.J. and G.H.Z. contributed to the interpretation of the numerical results and contributed to the writing of the manuscript. Y.Z.P. and G.Q.W contributed in the discussion. Z.H.Q and Q.W. participated in the numerical calculation. All authors reviewed the manuscript.

Additional information

Competing financial interests: The authors declare no competing financial interests.
